# ICU nursing connectivity and the quality of care in an academic medical center: a network analysis

**DOI:** 10.1186/cc13208

**Published:** 2014-03-17

**Authors:** DM Kelly, DC Angus, D Krackhardt, JM Kahn

**Affiliations:** 1 University of Pittsburgh, PA USA; 2 Carnegie Mellon University, Pittsburgh, PA, USA

## Introduction

A collaborative nurse work environment is associated with ICU quality, yet collaborative interaction is difficult to measure. Network analysis may be an innovative tool to measure interactions. We sought to determine the feasibility of network analysis to measure ICU nurse connectivity and test whether key network measures were associated with the ICU quality of care.

## Methods

We performed a network analysis in eight ICUs within an urban academic medical center in the United States during 2011. Using scheduling data, we defined network ties as instances when two ICU nurses worked together in the same ICU for 4 hours or more. We examined each ICUU+2019s network by visualizing sociograms and by measuring two network properties: density and clustering. Density measures the cohesion within a network on a scale from 0 to 100, with a higher score indicating more cohesion. Clustering assesses the local neighborhoods on a scale from 0 to 100, with a higher score indicating a more decentralized network. We examined variation in network measures across ICUs and tested the correlation between network measures and the proportion of patients receiving daily interruption of sedation (DIS).

## Results

**Figure 1:**
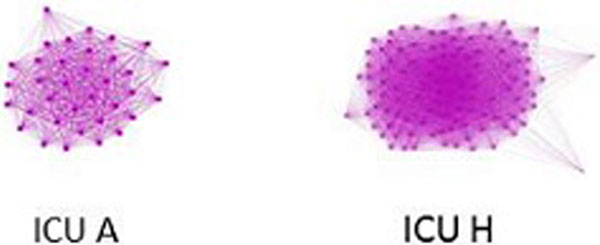
**Sociograms of ICU A and ICU H**.

There was wide variation in the networks, with density ranging from 79 to 96 and clustering ranging from 88 to 97. Two sample sociograms are shown in Figure 1: ICU A had a very high density (96) and clustering coefficient (97) suggesting a cohesive and decentralized network, contrasting with ICU H that had the lowest density (79) and clustering coefficient (88). Greater density and clustering was positively associated with DIS (B = 0.02 (-0.10, 0.14); B = 0.003 (-0.07, 0.07)) but did not achieve statistical significance in our small sample.

## Conclusion

We found substantial variation in ICU nursing networks across eight ICUs in one academic medical center. These patterns may have implications for evidence-based practice implementation. More work is needed to understand the role of network analysis as a reliable tool for improving and understanding ICU organization.

